# The Highly Expressed FAM83F Protein in Papillary Thyroid Cancer Exerts a Pro-Oncogenic Role in Thyroid Follicular Cells

**DOI:** 10.3389/fendo.2019.00134

**Published:** 2019-03-01

**Authors:** Cesar Seigi Fuziwara, Kelly Cristina Saito, Suzana Garcia Leoni, Ângela Flávia Logullo Waitzberg, Edna Teruko Kimura

**Affiliations:** ^1^Department of Cell and Developmental Biology, Institute of Biomedical Sciences, University of São Paulo, São Paulo, Brazil; ^2^Department of Pathology, Federal University of São Paulo, São Paulo, Brazil

**Keywords:** FAM83F, papillary thyroid cancer, thyroid cell differentiation, MAPK signaling, stem-cell genes

## Abstract

Thyroid cancer is the most common endocrine cancer with predominant prevalence of papillary thyroid cancer (PTC) histotype. MAPK signaling genetic alterations are frequent in PTC, affecting more than 80% of cases. These alterations constitutively activate MAPK signaling cross-regulating different pro-oncogenic pathways. However, additional molecular alterations associated with thyroid cancer are not completely understood. In this extent, the new family of proteins named FAM83 (FAMily with sequence similarity 83) was recently identified as mediator of oncogenic signaling in different types of cancer. Here we report FAM83F as a novel highly expressed protein in PTC. We evaluated FAM83F levels in 106 PTC specimens, 34 goiter, and 41 adjacent non-tumoral human thyroid, and observed FAM83F cytoplasmic overexpression in 71% of PTC (76 of 106) while goiter tissues showed nuclear positivity and normal thyroid showed no staining by immunohistochemistry. Moreover, TSH-induced goiter and *BRAF*^*T*1799*A*^-induced PTC animal models also showed FAM83F activation. *In vitro*, we generated a stable thyroid cell line PCCL3 with FAM83F overexpression and observed that FAM83F deregulates thyroid follicular cell biology leading to loss of thyroid differentiation genes such as Sodium-Iodide Symporter (NIS), reactivation of stem cell markers such as LIN28B and SOX2, induction of cell migration and resistance to doxorubicin-induced apoptosis. Moreover, FAM83F activates MAPK signaling through interaction with BRAF and RAF while impairs TGFβ antiproliferative signaling transduction. In this study, we showed FAM83F as a new pro-oncogenic protein overexpressed in thyroid cancer that modulates thyroid follicular cell biology and differentiation through cross-regulation of MAPK and TGFβ signaling.

## Introduction

Thyroid cancer is the most frequent endocrine cancer with rising incidence all over the world (1). Recent estimate shows that ~53,990 new cases of thyroid cancer will occur this year in the USA (2). The etiopathogenesis of papillary thyroid cancer (PTC), the most prevalent histotype (~80% of cases), is based on genetic alterations in the MAPK signaling pathway (i.e., RET, RAS, and BRAF) in ~80% of the cases, which display distinct clinical presentation (3–6). *BRAF*^*T*1799*A*^ is the most prevalent mutation in PTC, accounting for more than 40% of alterations detected (3). However, even BRAF-mutated PTC is a heterogeneous group with variable degrees of differentiation and clinical behavior (5, 7).

Loss of cell differentiation is associated with aggressive thyroid cancer as thyroid follicular cells lose Sodium-Iodide Symporter (NIS) expression and the ability to concentrate radioiodine which is often used as therapy after cancer resection (8, 9). NIS transports iodide from blood to thyroid cells which is oxidated by Thyroperoxidase (TPO) at the apical region and coupled to thyroglobulin (TG) at tyrosine residues, forming the precursors of thyroid hormones. The maintenance of thyroid differentiated status is exerted mainly by thyroid transcription factors TTF1 and PAX8 and the pituitary TSH (10).

Despite the current knowledge regarding thyroid oncogenesis, the identification of additional signaling pathways involved in thyroid oncogenesis and differential tumor behavior are still required. In this extent, a new family of proteins named FAM83 (FAMily with sequence similarity 83) comprising eight genes (FAM83A to H) was recently identified as mediators of oncogenic signaling in cancer (11). The classification of FAM83 proteins is based on the presence of the Domain of Unknown Function (DUF1669) in the N-terminus with putative phospholipase activity but lacking conservation at a critical histidine residue (HxKxxxxDxxxxxxIGSxN) present in all bona fide Phospholipase D (PLD) enzymes for catalytic activity (12). FAM83 members play an important role in cancer, acting to promote a more aggressive cell behavior in breast cancer and resistance to chemotherapy through MAPK signaling activation (13, 14). However, the role of FAM83 members is yet uncovered in thyroid cancer.

In this study, we identified FAM83F as a novel marker highly expressed in PTC which exerts a pro-oncogenic effect in thyroid cell behavior through modulating and interacting with MAPK and TGFβ pathways.

## Materials and Methods

### Thyroid Tumor Samples

Formalin-fixed paraffin embedded (FFPE) human thyroid tumors derived from total thyroidectomy were used in this study for immunohistochemical analyses. Tissues were removed upon patients' informed consent for the collection of biological samples. A subset of thyroid samples were collected in RNA*later*^TM^ and total RNA was extracted using Trizol^TM^ Reagent. This study complied with the guidelines from the human ethical committee of the Institute of Biomedical Sciences (CEPSH.250.09), University of São Paulo.

### Animals

Two-months female wistar rats were treated with 0.05% Methimazole (2-Mercapto-1-methylimidazole /MMI) (Sigma-Aldrich) in the drinking water for 5 days to induce pharmacological goiter by the increase of TSH serum levels. Then, thyroid tissue was surgically removed and fixed in 3.7% formaldehyde in PBS, and embedded in paraffin for immunohistochemical analyses.

FVB/N BRAF^T1799A^ transgenic mice (Tg-BRAF) develop PTC as the animals conditionally express BRAF^T1799A^ in thyroid follicular cells directed by thyroglobulin promoter (15). Thyroid tissue of 5-weeks mice was surgically removed and fixed in 3.7% formaldehyde in PBS, and embedded in paraffin for immunohistochemical analyses, and were compared with age matched normal FVB/N.

Animals were housed in controlled temperature (23 ± 2°C) and light cycle (12-h light/12 h dark), with food and water *ad libitum*. Animal euthanasia was performed using ketamine (500 mg/Kg IPs) and xylasine (50 mg/Kg IP) according to AVMA Guidelines for the Euthanasia of Animals. All procedures were conducted in accordance to the guidelines of Institutional Animal Care and Use Committee (IACUC) of the Institute of Biomedical Sciences, University of Sao Paulo (protocol number 108/2016).

### Cell Lines

Rat thyroid follicular cells PCCL3 (16) were used to establish stable cell line PCCL3-FAM83F by transfecting pCMV-neo-FAM83F (RC203874) containing Myc-DDK-tagged human FAM83F coding sequence (Origene Technologies, Rockville, MD). PCCL3-empty control cells (PCCL3-Ø) were established with empty plasmid and G418 selection. These cells were cultivated in F-12 Coon‘s Modification medium (Sigma, St. Louis, MO, USA) supplemented with 5% fetal bovine serum (FBS) and four hormones: 1 mU/ml bovine TSH (Sigma), 10 μg/ml insulin (Sigma), 5 μg/ml transferrin (Sigma), and 10 nM hydrocortisone (Sigma), 300 ug/ml G418 (Invitrogen, Carlsbad, CA).

Nthy-ori 3-1 derived from normal human primary thyroid follicular epithelial cells were purchased from ECAAC (European Collection of Cell Culture) and cultivated RPMI (Invitrogen) supplemented with 2 mM l-glutamine and 10% FBS. All cell lines were kept in humidified incubator at 37°C and 5% CO_2_.

### Cell Treatments

#### Doxorubicin

Induction of apoptosis was evaluated by the treatment of cells with 1 μM doxorubicin for 24 h and flow cytometry using Annexin V-FITC Kit (Invitrogen). After exposure to doxorubicin, cells were detached by trypsinization and pelleted with the supernatant. One hundred thousand cells were incubated with Annexin-V-FITC (Invitrogen) for 30 min and with propidium iodide (PI) for 30 min in Annexin-binding buffer. Green (Annexin V-FITC) and red (PI) fluorescence was accessed by counting 10,000 events under flow cytometry using Guava EasyCyte Mini (Millipore).

#### MAPK Signaling Inhibitor

MAPK signaling pathway was blocked with U0126 (Promega), a specific inhibitor of MEK1 and MEK2, at 5 μM concentration in the culture medium for 48 h. Total RNA and protein was extracted for measurement of thyroid differentiation genes expression.

### Immunohistochemistry (IHC)

FAM83F expression was detected by IHC using rabbit anti-FAM83F (N17) (sc-102517) (Santa Cruz Biotechnology) and/or anti-FAM83F N-terminal region (ARP52602_P050) (Aviva Systems Biology) in human thyroid samples: adjacent non-tumoral thyroid (*n* = 41), goiter (*n* = 34), and PTC (*n* = 106). Moreover, we performed FAM83F IHC in rat control thyroid (*n* = 5)/MMI treated (*n* = 5), and also 5-weeks FVB/N (*n* = 4) mice normal thyroid/5-weeks Tg-BRAF mice PTC (*n* = 5).

Briefly, 3 μm FFPE slices were deparafinated and hydrated in PBS. Endogenous peroxidase was blocked using 3.0% H_2_O_2_, and slides were incubated with anti-FAM83F antibody diluted 1:100 in TBS/BSA (vol/vol) overnight at 4°C. After proper wash in PBS, slides were incubated with secondary anti-rabbit biotinylated antibody (Sigma) diluted 1:250 in TBS/BSA for 2 h at room temperature, followed by washing and incubation with ExtrAvidin® peroxidase (Sigma) diluted 1:250 in TBS/BSA for 2 h at room temperature. After proper wash, slides were incubated with DAB/BSA in the presence of H_2_O_2_. Both FAM83F antibodies (N17 and ARP52602_P050) presented similar immunostaining, and immunopositivity was scored according to signal intensity as: negative (–), weak (+), medium (++), and strong (+++).

### Immunofluorescence

Twenty thousand PCCL3-ϕ and PCCL3-FAM83F cells were seeded into coverslips inserted in 24-well plates and cultivated for 48 h. Cell were pre-fixed by the addition of 500 uL of 3.7% formaldehyde direct to the culture medium for 3 min, followed by medium removal and fixation with 500 uL of 3.7% formaldehyde for 15 min on ice. After washing with PBS, coverslips were incubated with the primary antibody anti-LIN28 (Santa Cruz) and anti-SOX2 (Abcam) diluted in 1% BSA in PBS/ 0.2% Triton-X overnight at 4°C. Secondary anti-rabbit antibody conjugated with Alexa Fluor® 488 Goat Anti-Rabbit IgG (H+L) (Invitrogen) was incubated for 2 h at room temperature. For FFPE immunofluorescence assay, slides were processed as previously described in immunohistochemistry. After tissue hydration, primary anti-FAM83F and secondary incubations were performed as described for cells in coverslip. After washing, slides were incubated with 300 nM DAPI diluted in PBS for 30 min. Slides were mounted using UltraCruz^TM^ Mounting Medium (Santa Cruz Biotechnology) and fluorescence was accessed in a multiphoton confocal microscope Zeiss LSM-780 NLO (Carl Zeiss Microscopy, Jena, Germany) at the Core Facility for Scientific Research—University of Sao Paulo (USP) (CEFAP-USP).

### Quantitative Real-Time PCR

Gene expression was analyzed using total RNA phenol-chloroform extracted from cell lines using cell TRIzol^TM^ Reagent according to manufacturer instructions. Briefly, 3 ug of total RNA was reverse transcribed using M-MLV reverse transcriptase (Invitrogen) in the presence of oligo-dT primer. Next, quantitative PCR was performed using SYBR-Green Master Mix (LifeTechnologies) and specific primers (Supplemental Table 1) in ViiA7^TM^ Real-Time PCR System (LifeTechnologies), and gene expression was calculated using Q-Gene program (17).

### Luciferase Reporter Assays

To evaluate the activation of different signaling pathway, cells were transiently transfected with reporter plasmids p3TP-lux and SBE4-Luc (Addgene) for TGFβ signaling activation and pGL4.33 [luc2P/SRE/Hygro](Promega) for MAPK/ERK signaling activation. Moreover, to evaluate rat Nis and Tg promoter region activity, we used pNis 2.8 (18) and pGl-Tg (19) plasmids.

Briefly, 4 × 10^4^ cells seeded onto 24-well plate. After 72 h, cells were transfected with 300 ng of reporter plasmid plus 30 ng of pRL (Renilla luciferase) using Lipofectamine 2,000 (Invitrogen). Cell lysates were collected after 24 h of transfection, and luminescence was detected using Dual-Glo Luciferase Assay (Promega) in SpectraMax L2 luminometer (SpectraMax). Specifically for testing TGFβ signal responsiveness, PCCL3-ϕ and PCCL3-FAM83F cells were transfected with p3TP-lux and SBE4-Luc prior to the treatment with 1 ng/ml recombinant TGFβ1 (rTGFβ-Peprotech, Rocky Hill, NJ, USA) in culture medium for 24 h. For MAPK/ERK signaling analysis, cells were serum/hormones starved for 24 h before transfection of pGL4.33 [luc2P/SRE/Hygro], and after 2 h of serum addition, cell lysates were collected.

### Cell Viability Assay (MTT)

Cells were plated at 2 × 10^4^ cells /well in 96-well plates and cultured for 24 h. MTT (Invitrogen) was added to cell culture medium to a final concentration of 250 ug/ml and incubated at 37°C in 5% CO_2_ incubator for 4 h. Then, the medium was removed and 100 μL of isopropanol/ 0.01 M HCl was added to solute formazan crystals. Absorbance was measured at 595 nm in a plate spectrophotometer SpectraMax M (Molecular Devices).

### Cell Cycle

After a 24 h starving of FBS and hormones in the culture medium, cells were cultivated in the complete medium (FBS + TSH and other hormones) for another 24 h when cells were detached by trypsinization, pelleted with supernatant (dead cells), and fixed with 70% ethanol. After hydration in PBS, cells were treated with RNase (100 μg/ml) and DNA was stained with propidium iodide (50 μg/ml). Cell cycle was accessed by counting 10,000 events under flow cytometry using Guava EasyCyte Mini (Millipore).

### Cell Migration Assay

Migration assay was performed using uncoated Transwell chambers with membrane pore size of 8.0 μm (Corning Inc). Membranes were incubated with PBS during 1 h at 37°C, 5% CO_2_ atmosphere. About 2 × 10^4^ cells PCCL3-ϕ and PCCL3-FAM83F were suspended in culture medium containing 0.5% FBS and plated in the upper chamber, whereas the lower chamber contained culture medium with 5% FBS. After 24 h at 37°C, 5% CO_2_, non-migrating cells on the top chamber were removed using a cotton swab, and cells that migrated through the membrane were fixed in 4% paraformaldehyde diluted in PBS, and stained with 0.5% Crystal Violet. Cells were photographed using a Nikon Eclipse E600 microscope equipped with optical camera CF160 epi-fluorescence and counted (10 representative fields).

### Western-Blotting Analyses

Total protein was extracted from cells using RIPA buffer containing 10% protease inhibitor cocktail (Sigma, St. Louis, MO). Protein concentration was determined using Bradford (Bio-Rad Laboratories, Hercules, CA), and 40 μg of each sample was fractionated by 10–12% SDS-PAGE and blotted onto a nitrocellulose membrane (Hybond-ECL; Amersham Biosciences, Little Chalfont, UK). Non-specific binding sites were blocked with 5% non-fat dry milk in PBS−0.1% Tween-20. The following primary antibodies were used: anti-FAM83F (N17) (sc-102517), anti-p-ERK1/2 (sc-7393), anti-ERK1(sc-94), anti-BRAF (sc-166), anti-RAF-1 (sc-133), anti-Vimentin (sc-32322), anti-TTF-1 (sc-13040), anti PAX-8 (sc-81353), anti-β-actin (sc-47778) (Santa Cruz, Santa Cruz, CA, USA), anti-Myc-tag (TA100010) (OriGene Technologies), and anti-LIN28B (Cell Signaling). The anti-Nis antibody was kindly donated by Dr. Sissy Jhiang.

Immunoexpression was detected with horseradish peroxidase-conjugated secondary antibodies (GeHealthcare) and developed with luminol and p-cumaric acid (Sigma) reagents in the presence of H_2_O_2_. Chemoluminescence emission was visualized in an ImageQuant LAS4000 imaging system (GE Healthcare, Little Chalfont, UK).

### Immunoprecipitation (IP)

For immunoprecipitation, PCCL3-FAM83F and Nthy-ori-FAM83F cells were lysed in RIPA buffer containing 10% protease inhibitor cocktail and incubated with agarose-protein A/G beads (Santa Cruz) for pre-clearing. Next, cell lysates were incubated overnight at 4°C under agitation with 20 μL of the complex of agarose-protein A/G beads + 1 μg of one of the following antibodies: anti-Myc tag (OriGene Technologies), anti-BRAF (Santa Cruz), anti-RAF1 (Santa Cruz), or anti-HuR (Invitrogen). After the incubation, beads were pelleted at 1,000 × g at 4°C and washed in RIPA buffer. Beads were resuspended in 1 × Western Blotting loading buffer and denaturated at 95°C for 5 min before loading into 10% SDS-PAGE. Immunodetection was performed as already described in WB section.

### Bioinformatics and Data Access Banks

The prediction of FAM83F subcellular localization was performed online using LOCATE subcellular location database (http://locate.imb.uq.edu.au/) that compares four different programs: CELLO, pTarget, WoLFPSORT, and Multiloc. miRNA target prediction was performed using TargetScan. Moreover, protein interaction was searched at String database (https://string-db.org/) and Integrated Interactions Database (http://iid.ophid.utoronto.ca/iid/About/).

Data from The Cancer Genome Atlas (TCGA) was visualized using cBioportal website (www.cbioportal.org) (20), while miRNA data mining was performed as described previously (21).

### Statistical Analysis

The results were presented as the mean ± standard deviation (SD) and were submitted to analysis of variance followed by *t*-test or the Tukey test. Differences were considered significant at *P* < 0.05.

## Results

### FAM83F Is Overexpressed in Papillary Thyroid Cancer and Goiter

**Figure 1:**
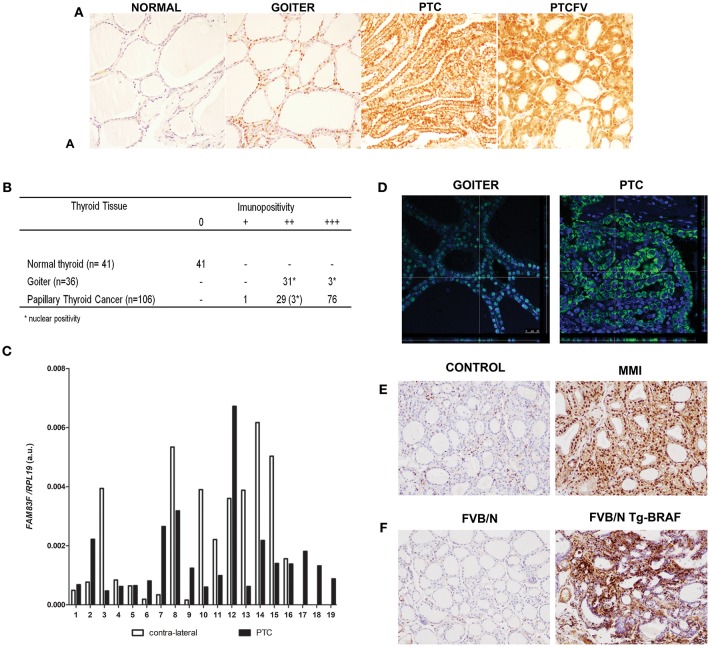
FAM83F protein expression in human thyroid tumors and in animal models. **(A)** Detection of FAM83F using IHC shows strong cytoplasmic expression in papillary thyroid cancer (PTC), while in goiter strong nuclear expression is detected. Normal adjacent thyroid tissue is mostly negative for FAM83F staining. **(B)** IHC quantification in PTC (*n* = 106), goiter (*n* = 34), and normal thyroid tissue (*n* = 41). **(C)** *FAM83F* gene expression in a set of 19 paired human PTC vs. contra-lateral thyroid tissue by qPCR. Data shown as relative expression of *FAM83F/RPL19*. **(D)** Confocal microscopy of FAM83F in goiter and PTC. Immunofluorescence staining of FAM83F protein in goiter section (left panel) shows nuclear localization observed in the z-stack that colocalizes the nucleus with the green fluorescence of FAM83F, while in PTC section (right panel) FAM83F expression is cytoplasmic. **(E)** FAM83F protein expression in rat-induced pharmacological goiter. The treatment of rats with MMI for 5 days increased immunopositivity of FAM83F in thyroid follicular cells with strong positivity in the nucleus and mild positivity in cytoplasm (*n* = 5). **(F)** FAM83F protein expression in *BRAF*^*T*1799*A*^ transgenic mice with PTC. Detection of FAM83F using IHC shows strong cytoplasmic and nuclear expression in 5–weeks old mice PTC (*n* = 5), while no expression is observed in normal thyroid (*n* = 4).

The analysis of FAM83F protein levels in human thyroid tumors revealed differential expression in malignant and goiter compared to normal thyroid follicular cells (Figure 1A). In normal thyroid tissues, FAM83F immunostaining was negative (0 of 41 positive), whereas all PTC were positive and 71% (76 of 106 positive) showed strong cytoplasmic staining independent of PTC variant. Moreover, human multi-nodular goiter tissue showed nuclear positivity in all cases (34 of 34 positive) (Figure 1B). Additionally, we analyzed *FAM83F* expression in a set of 19 paired PTC/ contra-lateral tissue and observed a variable mRNA levels (Figure 1C).

Confocal microscopy was used to confirm cytoplasmic expression of FAM83F in PTC (*n* = 15) and nuclear localization in goiter (*n* = 10). The confocal microscope z-stack showed that FAM83F was localized in the nucleus of human goiter cells and in the cytoplasm of PTC cells (Figure 1D). Similarly, we observed a predominant nuclear immunopositivity of FAM83F in thyroid tissue from pharmacological goiter induced in rats (Figure 1E). These animals were treated with methimazole (MMI) that blocks thyroid hormone production, induces TSH secretion and thyroid gland growth (thyroid weight-CTR: 15.35 ± 0.26 mg; MMI: 22.60 ± 2.52 mg. *P* value ≤ 0.01). Moreover, using the Tg-BRAF transgenic mouse model of PTC, we explored the *in vivo* influence of conditional *BRAF*^*T*1799*A*^ oncogene activation in FAM83F expression. Indeed, we observed Tg-BRAF-derived PTC had increased FAM83F positivity in the cytoplasm and nucleus compared to normal animals in 5-week-old mice (Figure 1F).

The *in silico* prediction of subcellular location of FAM83F protein using the LOCATE subcellular location database (http://locate.imb.uq.edu.au/) showed a stronger prediction for the nucleus in four programs (CELLO, pTarget, WoLFPSORT, and Multiloc) but also a cytoplasmic location in two programs (CELLO and WoLFPSORT).

Interestingly, data from TCGA accessed at the cBioportal website shows that PTC is among the top 10 types of cancer with higher levels of *FAM83F* (5, 20) (Supplemental Figure 1A). Using a larger cohort of 60 PTC patients data extracted from TCGA, we observed that FAM83F mRNA is slightly down-regulated in PTC suggesting the influence of post-transcriptional modifications that could enhance FAM83F protein levels in PTC (Supplemental Figure 1B). Thus, we analyzed expression of *miR-143* and, miRNAs that potentially target *FAM83F* 3′-UTR and blocks its translation, and observed significant down-regulation of these miRNAs in TCGA PTC samples (Supplemental Figures 1C,D).

### FAM83F Overexpression Alters Thyroid Follicular Cell Differentiation Genes

**Figure 2:**
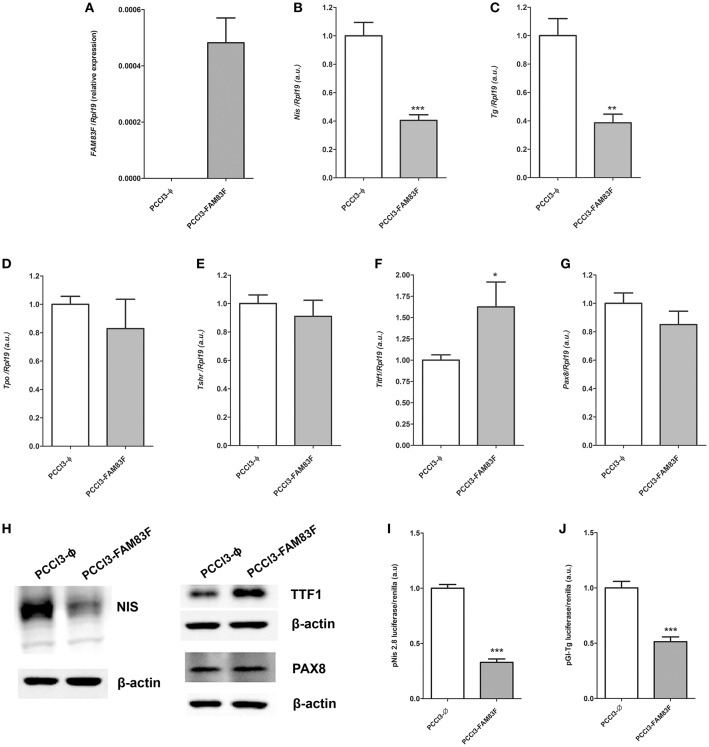
FAM83F overexpression effects on thyroid differentiation genes of PCCL3 cell line. **(A)** Validation of *FAM83F* gene expression increase in PCCL3-FAM83F. In this case, data shown as relative expression due to undetectable levels of *FAM83F* in PCCL3-ϕ by qPCR; Expression levels of **(B)** *Nis;* **(C)**: *Tg*; **(D)** *Tpo*; **(E)** *Tshr*; **(F)** *Ttf1*, and **(G)** *Pax8* in PCCL3-FAM83F cells by qPCR. Data shown as mean ± SD of two independent experiments performed in triplicate normalized by PCCL3-ϕ. ^*^P < 0.05, ^**^P < 0.01 and ^**^P < 0.001. **(H)** Protein expression of NIS, TTF1 (NKX2-1), and PAX8 analyzed by Western-blotting in PCCL3-FAM83F cells. **(I)** Transcriptional activity of Nis promoter measured after transfection of a reporter assay with pNis 2.8 luciferase plasmid; and **(J)** Tg promoter activity measured with pGl-Tg luciferase plasmid into PCCL3-FAM83F cells compared to PCCL3-ϕ. Data shown as mean ± SD of two independent experiments performed in triplicate. ^**^P < 0.01 and ^***^P < 0.001.

In order to investigate the role of FAM83F in thyroid cell biology, we constructed a FAM83F overexpression stable cell line (PCCL3-FAM83F) derived from normal thyroid follicular cell PCCL3 via plasmid transfection (Figure 2A). As result, we observed high levels of *FAM83F* mRNA (Figure 2A), and FAM83F protein is localized predominantly in the cytoplasm compared to empty control (PCCL3-Ø) (Supplemental Figure 2A). We first analyzed the influence of FAM83F overexpression on thyroid cell differentiation. The expression of the iodide-metabolizing genes *Nis* and *Tg* in PCCL3-FAM83F cells was reduced to ~40% of control cell levels (Figures 2B,C), while no changes were observed for *Tpo* and *Tshr* (Figures 2D,E). Moreover, surprisingly, thyroid transcription factor *Ttf1* expression was increased with FAM83F overexpression, while no changes were observed in *Pax8* levels (Figures 2F,G).

Indeed, NIS protein downregulation was observed in PCCL3-FAM83F by Western blotting assay (Figure 2H). Interestingly, TTF1 levels were increased, while no changes were observed in PAX8 protein in PCCL3-FAM83F cells. In order to investigate the effect of FAM83F on *Nis* and *Tg* genes transcription, we used Nis and Tg promoter luciferase reporters that contain the promoter region of *Nis* (pNis 2.8) and *Tg* (pGl-Tg) genes upstream of the firefly luciferase gene. We observed that *Nis* and *Tg* promoter activity was reduced in response to FAM83F overexpression (Figures 2I,J). pNis 2.8 contains 2854-bp DNA fragment of rat *Nis* promoter that encompass one TTF1 binding site and two Pax-8 binding sites. pGL-Tg contains the upstream proximal promoter of *TG* (−180 to +22 relative to transcription start site) that encompass three TTF1 binding sites, one TTF2 binding site and one PAX8 binding site.

### FAM83F Overexpression Enhances the Stem-Cell Phenotype and Proliferation

**Figure 3:**
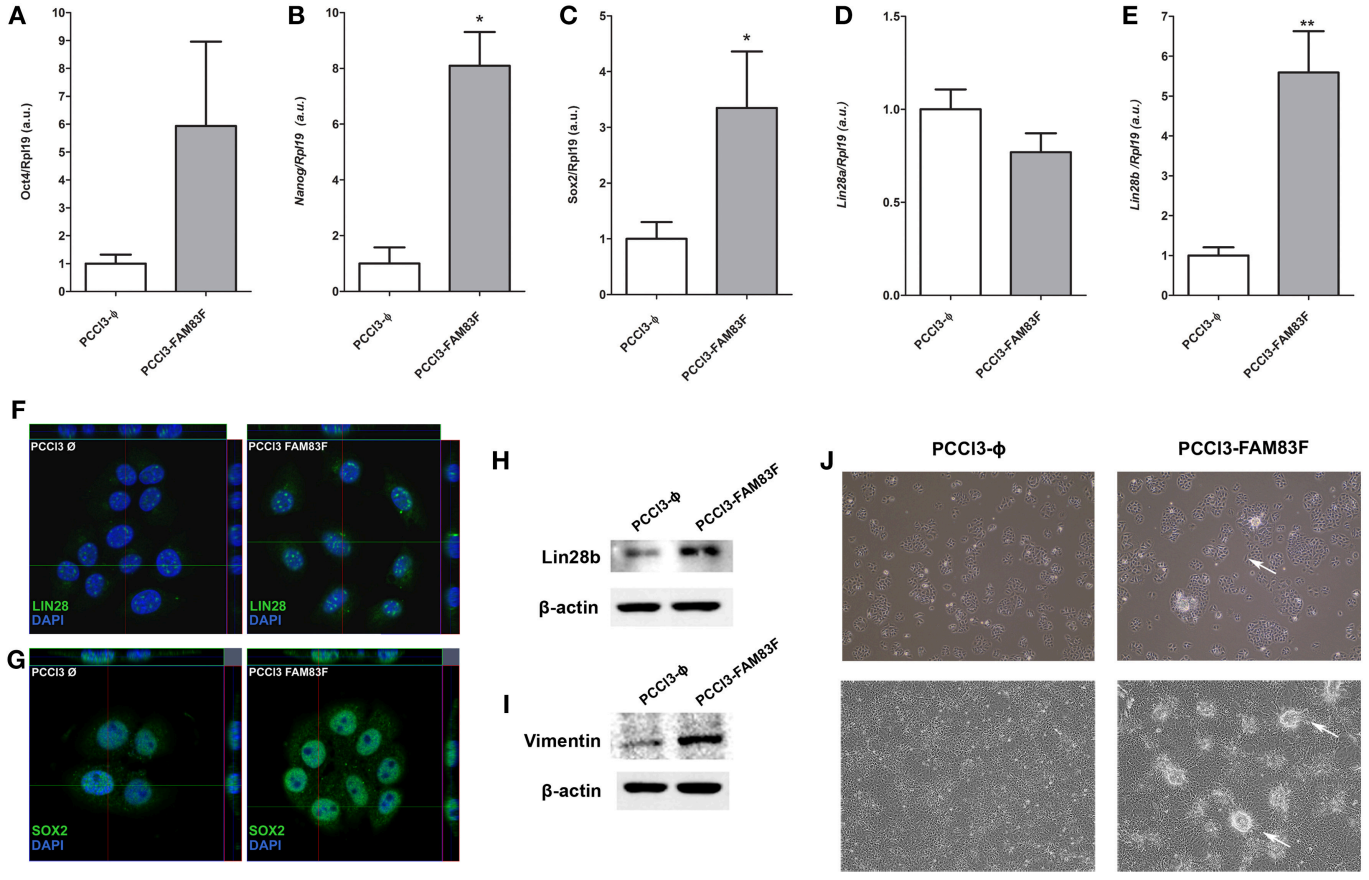
FAM83F overexpression effects on stem cell markers expression of PCCL3 cell line. **(A)** Gene expression of *Oct4*; **(B)** *Nanog*; **(C)** *Sox2*; **(D)** *Lin28a*; and **(E)** *Lin28b* by qPCR in PCCL3-FAM83F cells. Data shown as mean ± SD of two independent experiments performed in triplicate normalized by PCCL3-ϕ. ^*^P < 0.05; ^**^P < 0.01. **(F,G)** Protein expression of LIN28 and SOX2 analyzed by confocal microscopy in PCCL3-FAM83F cells using secondary antibody anti-rabbit Alexa Fluor®488 (green); **(H)** LIN28B protein levels and; **(I)** Vimentin protein levels in PCCL3-FAM83F compared to PCCL3-ϕ by Western-blotting. The same control β-actin was used from Figure 2H as membranes were probed with different antibodies. **(J)** Cell morphology of PCCL3-FAM83F and PCCL3-ϕ cells in culture at low and high confluency, showing the formation of spherical structures (indicated by the arrows) in PCCL3-FAM83F. 10 × magnification under EVOS® XL Core Imaging system.

Allied to the loss thyroid differentiation markers expression, we analyzed the expression of *Oct4, Sox2, Nanog, Lin28a*, and *Lin28b*, genes related to the “stemness” phenotype, and observed increased gene expression of *Sox2, Nanog* and *Lin28b* in PCCL3-FAM83F compared to control cells (Figures 3A–E).

Increased protein expression of LIN28B and SOX2 was also demonstrated in PCCL3-FAM83F by confocal immunofluorescence or Western blotting (LIN28B) (Figures 3F–H). Moreover, the protein level of vimentin, a mesenchymal marker, was increased with FAM83F overexpression (Figure 3I).

**Figure 4:**
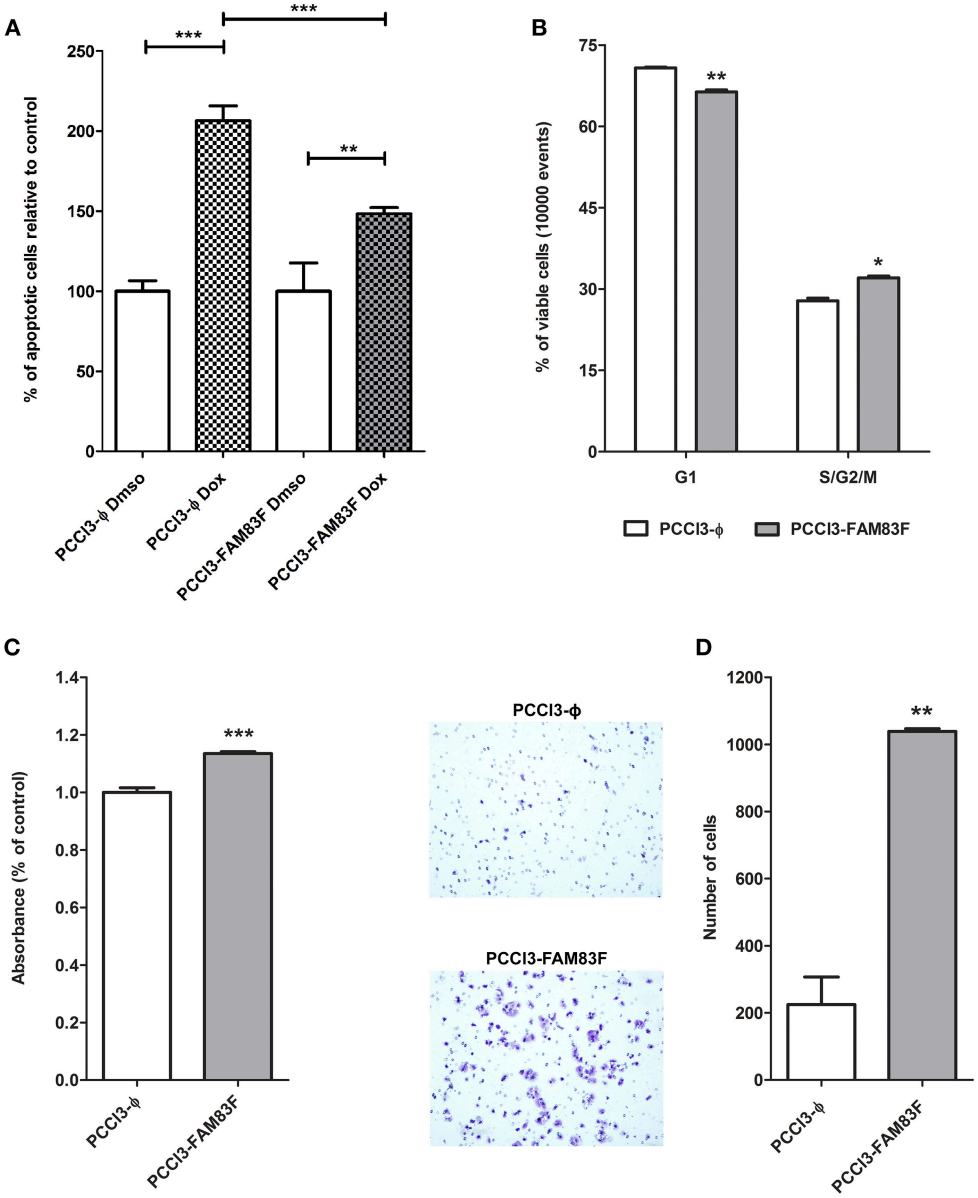
FAM83F overexpression effects on PCCL3 cells biology. **(A)** Apoptosis levels in response to doxorubicin treatment. PCCL3-FAM83F and PCCL3-ϕ cells were treated with doxorubicin for 24 h and apoptosis was detected as Annexin-V-FITC positivity. Representative results of two independent experiments performed in triplicate. **(B)** Cell cycle analysis of PCCL3-FAM83F and PCCL3-ϕ cells performed by flow-cytometry with PI staining. ^*^P < 0.05, ^**^P < 0.01, and^**^P < 0.001. **(C)** PCCL3-FAM83F and PCCL3-ϕ cell viability was measured by MTT-assay. Data shown as mean ± SD of two independent experiments performed in triplicate. **(D)** Cell migration analysis of PCCL3-FAM83F and PCCL3-ϕ measured by 8 μm-pore transwell assay. Representative image of two independent experiments performed in triplicate. ^*^P < 0.05, ^**^P < 0.01, and ^**^P < 0.001.

Indeed, we observed that PCCL3-FAM83F cultures showed the presence of particular globular (spherical) structures in low and high confluency indicative of multilayer growth and loss of differentiation (Figure 3J). These morphological changes in PCCL3-FAM83F cultures along with the expression of “stemness” markers led us to investigate responsiveness to the chemotherapeutic drug doxorubicin, as resistance is commonly observed in cancer stem cells population (22). Indeed, PCCL3-FAM83F cells were more resistant to the cytotoxic effects of doxorubicin, as observed by the fewer apoptotic cells compared to the control group (PCCL3-Φ) (Figure 4A). FAM83F overexpression did not alter the baseline level of apoptotic cells compared to PCCL3-Φ.

The analysis of the cell cycle showed a reduction in G1 phase and increase in S/G2/M fraction of PCCL3-FAM83F cells compared to PCCL3-ϕ cells (Figure 4B). The MTT assay showed that FAM83F overexpression in PCCL3 cells increased cell viability by 15% (Figure 4C). Moreover, PCCL3-FAM83F cells showed enhanced migration through pores (5-fold increase) in the transwell assay (Figure 4D), and in a wound-assay compared to control (Supplemental Figure 2B).

**Figure 5:**
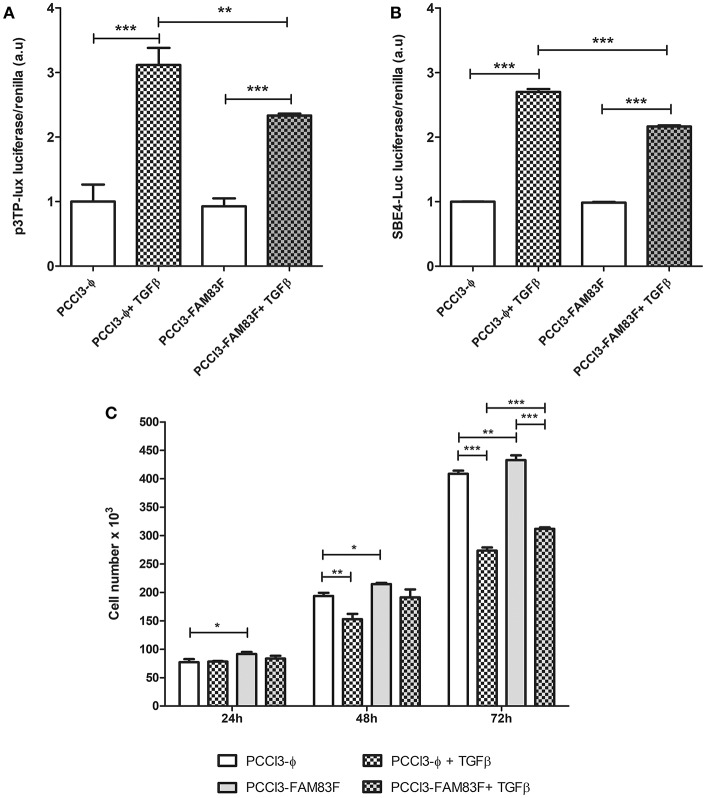
FAM83F overexpression effect on TGFβ signaling in PCCL3 cells. **(A,B)** Responsiveness of TGFβ signaling was analyzed 24-h after the co-transfection of p3TP-lux and 4 × SBE luciferase reporter plasmids with pRl (Renilla luciferase) plasmid and treatment with 1 ng/ml TGFβ in PCCL3-FAM83F and PCCL3-ϕ cells. **(C)** Cell counting assay in response to rTGFβ1. Cells were seeded at the density of 5 × 10^4^ cell/ well in 6-well plate and treated with 1 ng/ml of rTGFβ1 for 24–72 h and counted in a Guava minicytometer (Millipore). Data shown as mean ± SD of two independent experiments performed in triplicate. ^*^P < 0.05, ^**^P < 0.01 and ^***^P < 0.001.

One of the negative regulators of normal thyroid follicular cells proliferation is TGFβ pathway. Accordingly, we used luciferase reporter plasmids (3-TPlux and 4 × SBE) containing elements responsive to the activation of TGFβ pathway. We observed that PCCL3-FAM83F exhibited a reduction in responsiveness to rTGFβ treatment using the p3TP-lux and pSBE4-Luc plasmids containing four Smad-binding elements (SBE) (Figures 5A,B). Indeed, we also observed growth advantage in FAM83F cells in cell counting soon after 24 h of plating until 72 h (Figure 5C), which corroborated with our previous data from cell cycle enhancement. Interestingly, even in the presence of rTGFβ1, an antimitogenic factor, PCCL3-FAM83F have increased cell number, which corroborates the loss of TGFβ signaling responsiveness (Figure 5C).

### FAM83F and MAPK Signaling

**Figure 6:**
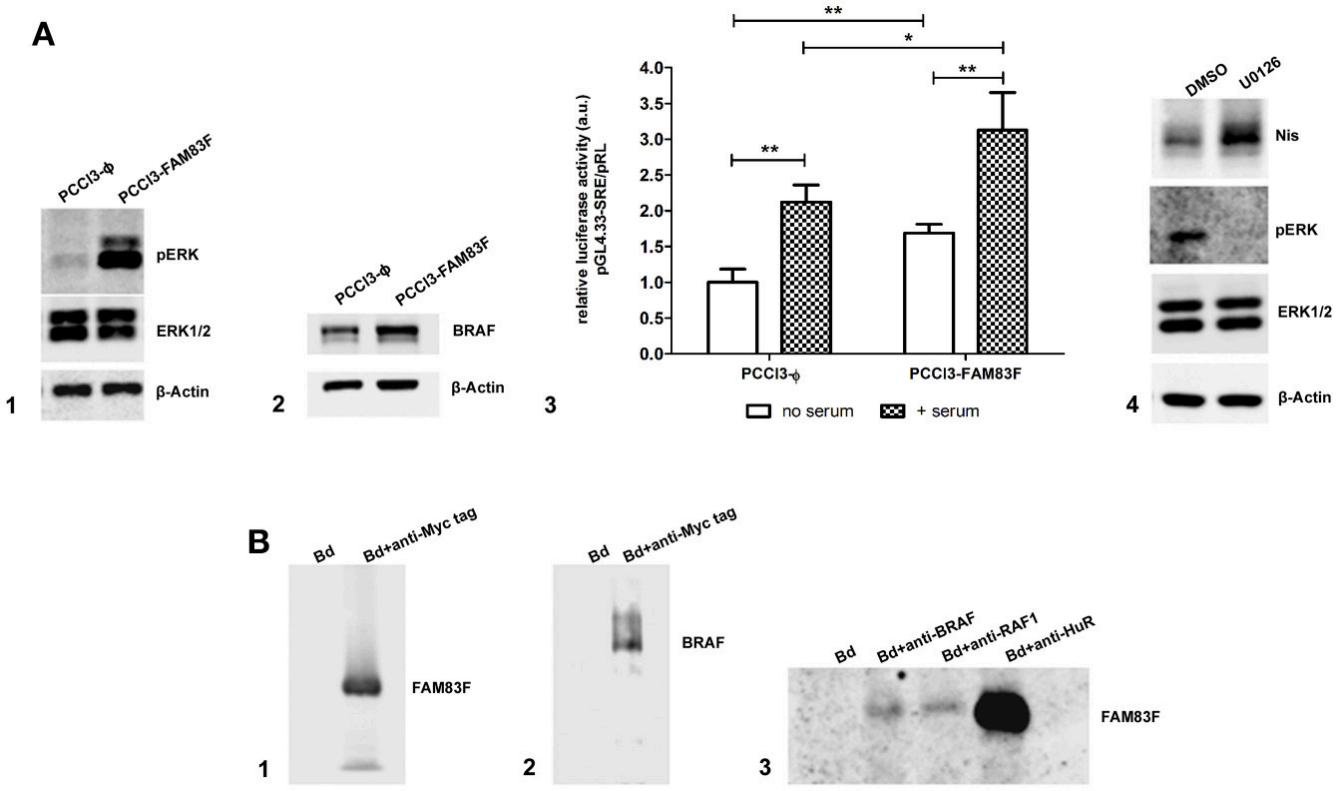
FAM83F overexpression effect on MAPK signaling. **(A)** Analysis of MAPK signaling components protein expression and activation in rat thyroid follicular cells PCCL3 cells overexpressing FAM83F; **(A1)** Western-blotting of pERK and ERK1/2 levels; **(A2)** Western-blotting of BRAF protein levels; **(A3)** luciferase reporter assay with the pGL4.33[luc2P/SRE/Hygro] plasmid that contains serum-responsive elements activated by MAPK/ERK signaling in PCCL3-FAM83F after 2 h of serum addition to culture medium. Results are shown as mean normalized values (luminescence of pGL/ luminescence of pRL plasmid). ^*^P < 0.05 and ^**^P < 0.01. **(A4)** Western-blotting of Nis in PCCL3-FAM83F cells treated with MAPK signaling inhibitor U0126 at 5 uM for 48 h. **(B)** Immunoprecipitation (IP) in PCCL3-FAM83F cells using a anti-Myc Tag antibody to immunoprecipitate FAM83F protein (Myc-tagged); **(B1)** Detection of FAM83F protein levels in Myc-Tag IP lysate by WB; **(B2)** Detection of BRAF protein levels in Myc-Tag IP lysate by WB; **(B3)** Immunoprecipation of BRAF, RAF1, and HuR followed by detection of FAM83F in PCCL3-FAM83F cells by WB. Bd group stands for beads only IP (no antibody).

Next, we analyzed the protein expression of MAPK/ERK signaling which regulates cell proliferation and differentiation, and we observed that FAM83F increased BRAF levels and induced the activation of pERK (Figures 6A1,A2). Moreover, a functional luciferase reporter assay confirmed the activation of MAPK/ERK pathway even in the absence of serum using pGl4-SRE reporter plasmid (Figure 6A3).

Treatment of PCCL3-FAM83F cells with U0126 (5 μM for 48 h), a specific MEK1/2 inhibitor, increased Nis protein levels (Figure 6A4). However, interestingly, this effect was also observed in control cells that showed increased NIS levels in control (Supplemental Figure 2C). MAPK inhibition with U0126 suppressed FAM83F effect over NIS expression indicating that blockade of FAM83F-induced MAPK/ERK activation is sufficient to prevent NIS downregulation (Supplemental Figure 2C).

Previous studies with other isoforms of FAM83 proteins have shown that these proteins interact with MAPK signaling components such as RAF-1 (13, 14). In this extent, we investigated the interaction of FAM83F with cytoplasmic proteins performing an immunoprecipitation (IP) assay for FAM83F using an anti-Myc-tag antibody fused to the protein in PCCL3-FAM83F cells (Figure 6B1). IP revealed that BRAF and RAF1 co-immunoprecipitated with FAM83F in PCCL3-FAM83F cells (Figures 6B2,B3). Moreover, *in silico* search in String Database and Integrated Interactions Database showed that FAM83F could interact with ELAVL1 (HuR) protein. Here we demonstrated that the RNA-binding protein HuR interacted with FAM83F in PCCL3-FAM83F cells (Figure 6B3). Indeed, we also detected this interaction with BRAF, RAF1 and HuR in Nthy-ori 3–1 cell line derived from human thyroid follicular cells when FAM83F is overexpressed (Supplemental Figure 2D).

## Discussion

The FAM83 family of proteins comprises novel and not yet completely characterized proteins with pro-oncogenic effects. Recent studies have shown an oncogenic role for FAM83 family proteins. FAM83A, originally identified as BJ-TSA-9, is overexpressed in lung cancer and breast cancer tumors, and it is associated with breast cancer refractoriness to EGFR-TKI (tyrosine kinase inhibitors) (14, 23). FAM83B is overexpressed in breast and lung cancer, and other types of solid cancers, including those of the bladder, cervix, ovary, and testis (13). FAM83D is overexpressed in breast and hepatocellular cancer (24, 25), while FAM83F is overexpressed in esophageal cancer (26). Moreover, a recent study reports correlation between FAM83F copy number and gene expression in thyroid carcinoma (27).

In our study, we showed that FAM83F was overexpressed in PTC with a strong cytoplasmic immunopositivity, differing from goiter, which displayed a predominantly nuclear expression. On the other hand, the normal thyroid follicles were essentially negative. The *in vivo* model of pharmacologically induced goiter in rats indicated an important role for TSH signaling in FAM83F expression and subcellular location, predominantly nuclear, which differed drastically from that in PTC. Moreover, using a *BRAF*^*T*1799*A*^ transgenic mouse model, we also showed FAM83F overexpression in *BRAF*^*T*1799*A*^–induced PTC with nuclear and cytoplasmic positivity. Tg-BRAF mice show increase TSH levels due to hypothyroidism (15), and this effect could explain the nuclear expression of FAM83F in Tg-BRAF PTC samples.

The clear shift from nuclear to cytoplasmic accumulation in malignant compared to goiter indicates that the interaction of FAM83F with cytoplasmic proteins mediates the activation of different signaling pathways contributing to thyroid oncogenesis. Among known FAM83F interactors are ELAVL1 (ELAV-like RNA-binding protein, also known as HuR) and MME (membrane metallo-endopeptidase, a transmembrane glycoprotein). HuR is a RNA-binding protein that can shuttle from cytoplasm to nucleus due to a sequence that contains both NLS (Nuclear localization signal)/NES (Nuclear export signal) activity (28). Indeed, HuR is overexpressed in human thyroid tumors and also displays distinct nuclear-cytoplasmic localization, being the cytoplasmic expression associated to malignant phenotype in PTC (29). Interestingly, the similar pattern of expression of FAM83F and HuR proteins in thyroid tumors indicates that the interaction between these proteins, as we showed by our immunoprecipitation assay, may have a role in thyroid tumorigenesis which needs further investigation in future studies. Recently, a study showed that all FAM83 family protein members interact with Casein Kinase 1 (CSKN1) isoforms via DUF1669 domain indicating that this interaction may also modulate FAM83 function (30).

Functional analysis of FAM83F revealed a pro-oncogenic effect in normal thyroid follicular cells associated with the loss of thyroid cell differentiation markers such as NIS. The blunting of NIS expression is associated with the development of refractoriness to radioiodine in aggressive PTC (31). Moreover, we observed that FAM83F overexpression induced the reactivation of stem cell markers such as *Sox2, Nanog* and *Lin28b* usually associated cancer stem cells and undifferentiated thyroid cancer (32). This stem cell phenotype is also associated with resistance to chemotherapy as we observed in our results with thyroid follicular cells treated with doxorubicin, and it is also linked to increased cell viability and migration.

The conservation of the N-terminal DUF1669 among the eight members of this family (FAM83A to FAM83H) suggests a similar mechanism of action (12). DUF1669 contains a putative phospholipase D like domain that does not exhibit phospholipase catalytic activity due to the lack of conservation at a critical histidine residue (HxKxxxxDxxxxxxIGSxN) in all FAM83 isoforms, except FAM83D, present in all bona fide PLD enzymes for catalytic activity, and experimentally proved for FAM83A and FAM83B (13, 14). Despite this fact, DUF1669 domain seems to be the key part of FAM83 proteins effect since the deletion of this domain in FAM83B abrogates its effect in colony formation and ERK activation (13).

Overexpression of FAM83A, FAM83B, and FAM83D has been shown to activate MAPK signaling (13, 14, 24). Indeed, our results show that induction of FAM83F leads to MAPK signaling activation in thyroid follicular cells. The DUF1669 domain is necessary for the interaction of FAM83B with RAF1 (13), which is also observed for FAM83A (14). Such interaction of RAF1 with FAM83F was also observed in our study, and we found a new interaction with BRAF protein, demonstrating the participation of FAM83F in MAPK signaling pathway in thyroid cells. Indeed, it is well-stablished that MAPK signaling activation leads to downregulation of thyroid differentiation markers such as NIS (33–36). Our results show that FAM83F indirectly reduces NIS protein levels in PCCL3-FAM83F cells by activation of MAPK signaling.

We also examined the dysregulation of antiproliferative pathway and observed that FAM83F activation results in impairment of TGFβ signaling transduction and cell growth in response to TGFβ treatment. In thyroid follicular cells, TGFβ signaling is an important antiproliferative pathway, which is commonly deregulated in thyroid cancer and is associated with epithelial-to-mesenchymal transition in aggressive cancer (37–39). Remarkably, the activation of *BRAF*^*T*1799*A*^ oncogene mimics FAM83F effects observed over TGFβ signaling transduction (36, 40).

A prominent role for BRAF rather than RAF1 in thyroid cell biology has been reported as BRAF is essential for ERK phosphorylation and MAPK signaling activation (41). Indeed, genetic alteration in BRAF (*BRAF*^*T*1799*A*^) is the most frequent event that leads to thyroid cancer development (3, 15), indicating the importance of BRAF protein in thyroid cell oncogenesis. We also showed that *BRAF*^*T*1799*A*^-induced thyroid oncogenesis model *in vivo* (Tg-BRAF transgenic mice) increased FAM83F expression in 5-week-old PTC mice, altogether indicating a cross regulation of FAM83F and MAPK signaling.

As FAM83F protein was highly expressed in PTC while mRNA changes were not correlated in PTC samples, miRNA mediated post-transcriptionally regulation was investigated. miRNA target prediction with TargetScan showed that FAM83F mRNA 3′-UTR contains multiple binding sites for miRNAs highly expressed in normal thyroid, such as miR-143, miR-125b, miR-29a, miR-30a, miR-30c, miR-30d, and miR-22 (42), indicating that downregulation of these miRNAs in thyroid cancer would potentially increase FAM83F protein levels. Indeed, a recent study showed that in esophageal cancer, FAM83F is targeted by miR-143 (26). Interestingly, miR-143 is downregulated in PTC (43) and in our TCGA extracted cohort (Supplemental Figures 1C,D), as well as miR-455, another miRNA that controls FAM83F (44), suggesting that a post-transcriptional effect by miR-143/ miR-455 would increase FAM83F protein in PTC.

This study showed that FAM83F is overexpressed in PTC and that its overexpression in normal thyroid cells plays a pro-oncogenic role by leading to thyroid cell dedifferentiation (especially loss of Nis expression), activating thyroid cell migration and causing resistance to chemotherapy. This effect is associated with the activation of MAPK and cross-talk with TGFβ signaling. Thus, FAM83F is a novel marker of PTC, and further investigation is necessary to uncover the potential of FAM83F inhibition as a molecular target for thyroid cancer.

## Data Availability

All datasets generated for this study are included in the manuscript and/or the supplementary files.

## Author Contributions

CF performed most of the experiments. KS performed the immunofluorescence and data analysis. SL performed the real-time PCR and *in silico* analysis. ÂW participated in anatomopathological selection of human thyroid tissue. CF and EK conceived this study, analyzed the data, and wrote the manuscript with consultation of all authors.

### Conflict of Interest Statement

The authors declare that the research was conducted in the absence of any commercial or financial relationships that could be construed as a potential conflict of interest.

## Supplementary Material

Supplemental Figure 1 **(A)** FAM83F gene expression in different types of cancer extracted from TCGA database via cBioportal website. **(B)** FAM83F gene expression; **(C)** miR-143, and **(D)** miR-455 gene expression in a cohort of 60 PTC patients extracted from TCGA database as described previously (21). ^*^*P* < 0.01 vs. non-tumoral, ^***^*P* < 0.001 vs. non-tumoral using Mann-Whitney test.

Supplemental Figure 2 **(A)** Imunodetection of FAM83F using an anti Myc-tag antibody and secondary antibody conjugated with Alexa Fluor 488 by imunofluorescence. Green fluorescence indicates the presence of FAM83F expression. **(B)** Wound-healing assay for migration analysis: Cell migration was enhanced in PCCL3-FAM83F compared to PCCL3-Φ cells shown by wound closure after 8 days of culture in complete medium. Representative image from two independent experiments performed in triplicate. 10 × magnification under EVOS® XL Core Imaging system. **(C)** Nis protein expression after MAPK signaling inhibition with U0126 at 5 μM for 48 h in PCCL3-FAM83F compared to PCCL3-Φ cells. **(D)** Analysis of MAPK signaling components in human non-tumoral thyroid follicular cells Nthy-ori 3–1 cells overexpressing FAM83F; **(D1)** Detection of BRAF protein in anti-Myc-Tag immunoprecipitated lysate from Nthy-ori-FAM83F cells by WB; **(D2)** Detection of RAF1 protein levels in anti-Myc-Tag IP lysate from Nthy-ori-FAM83F cells by WB; **(D3)** Detection of FAM83F protein levels in anti-Myc-Tag IP and anti-HuR immunoprecipitated lysate from Nthy-ori-FAM83F cells by WB. Bd group stands for beads only IP (no antibody).

Supplemental Table 1 Oligonucleotides used for qPCR.

## Abbreviations

FAM83: *F*AMily with sequence similarity 83
NIS: Sodium Iodide Symporter
MAPK: Mitogen-Activated Protein Kinase
PTC: Papillary Thyroid Cancer
DUF: Domain of Unknown Function.
