# Supplementary figures and images for: The Highly Expressed FAM83F Protein in Papillary Thyroid Cancer Exerts a Pro-Oncogenic Role in Thyroid Follicular Cells
Source: Front Endocrinol (Lausanne). 2019 Mar 1;10:134. doi: 10.3389/fendo.2019.00134 (PMC6407429; doi:10.3389/fendo.2019.00134)

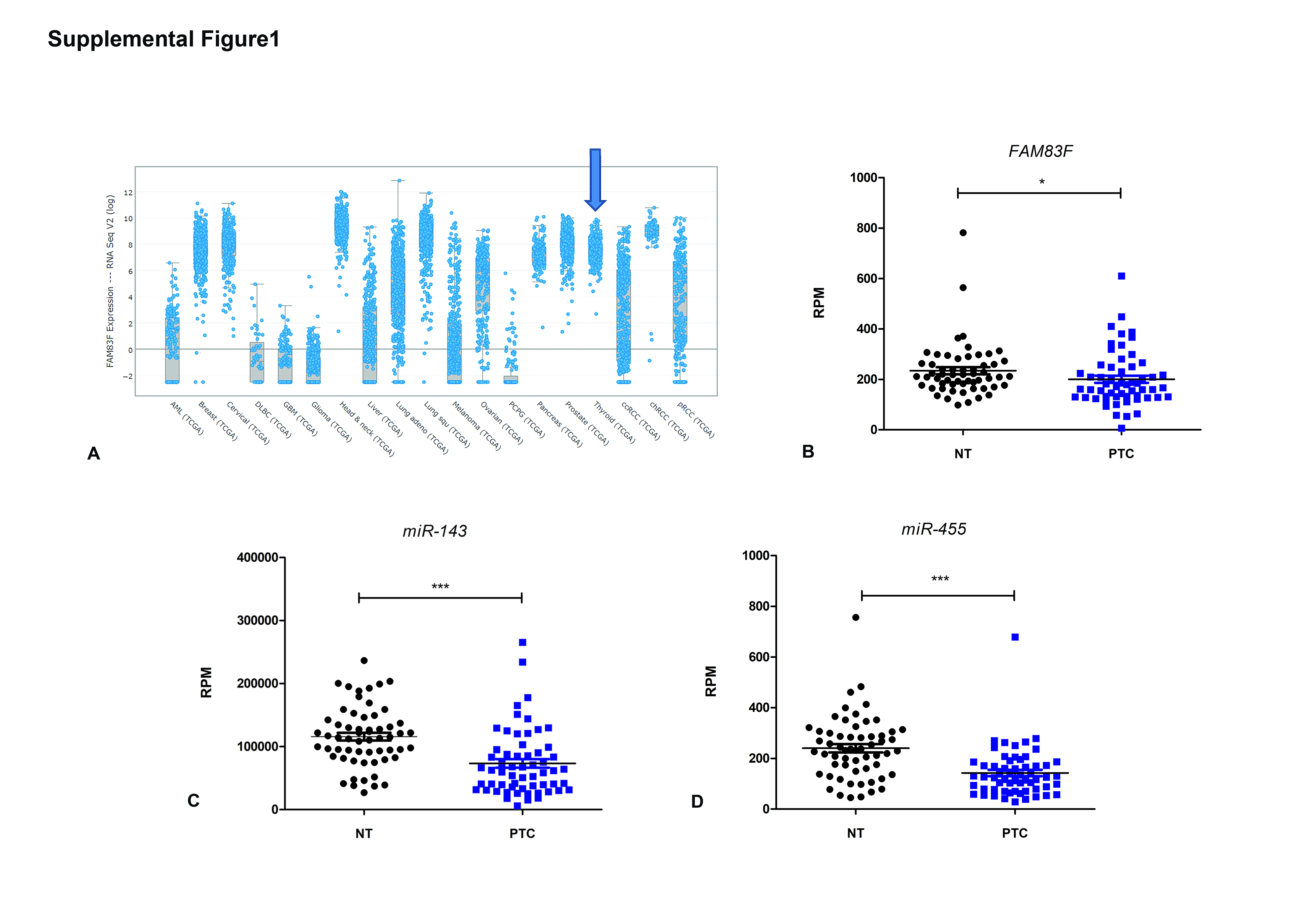

Supplement: Supplemental Figure 1 — (A) FAM83F gene expression in different types of cancer extracted from TCGA database via cBioportal website. (B) FAM83F gene expression; (C) miR-143, and (D) miR-455 gene expression in a cohort of 60 PTC patients extracted from TCGA database as described previously (21). *P < 0.01 vs. non-tumoral, ***P < 0.001 vs. non-tumoral using Mann-Whitney test. [file Image_1.TIF]

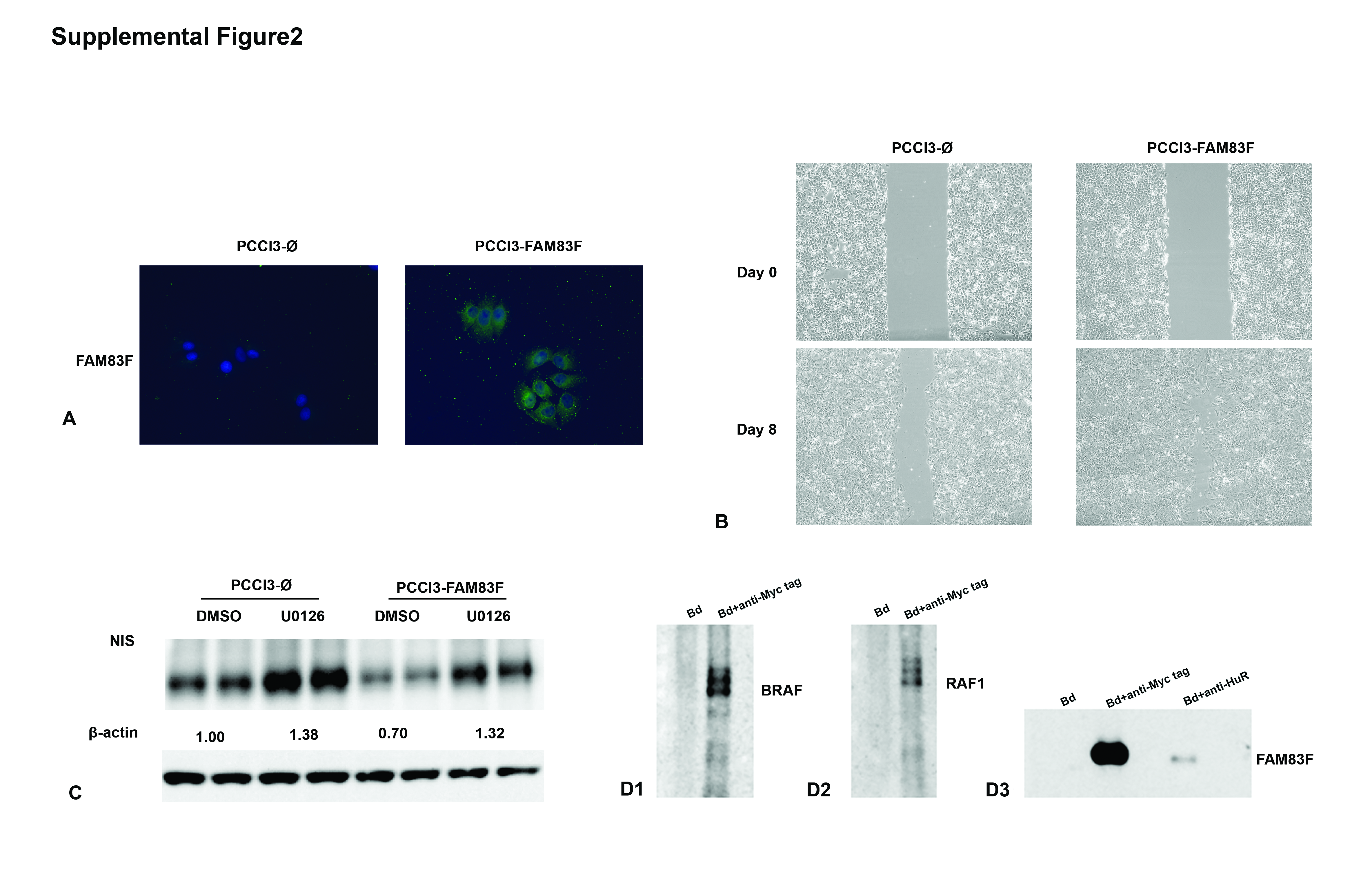

Supplement: Supplemental Figure 2 — (A) Imunodetection of FAM83F using an anti Myc-tag antibody and secondary antibody conjugated with Alexa Fluor 488 by imunofluorescence. Green fluorescence indicates the presence of FAM83F expression. (B) Wound-healing assay for migration analysis: Cell migration was enhanced in PCCL3-FAM83F compared to PCCL3-Φ cells shown by wound closure after 8 days of culture in complete medium. Representative image from two independent experiments performed in triplicate. 10 × magnification under EVOS® XL Core Imaging system. (C) Nis protein expression after MAPK signaling inhibition with U0126 at 5 μM for 48 h in PCCL3-FAM83F compared to PCCL3-Φ cells. (D) Analysis of MAPK signaling components in human non-tumoral thyroid follicular cells Nthy-ori 3–1 cells overexpressing FAM83F; (D1) Detection of BRAF protein in anti-Myc-Tag immunoprecipitated lysate from Nthy-ori-FAM83F cells by WB; (D2) Detection of RAF1 protein levels in anti-Myc-Tag IP lysate from Nthy-ori-FAM83F cells by WB; (D3) Detection of FAM83F protein levels in anti-Myc-Tag IP and anti-HuR immunoprecipitated lysate from Nthy-ori-FAM83F cells by WB. Bd group stands for beads only IP (no antibody). [file Image_2.TIF]
