# Supplementary material for: The Highly Expressed FAM83F Protein in Papillary Thyroid Cancer Exerts a Pro-Oncogenic Role in Thyroid Follicular Cells
Source: Front Endocrinol (Lausanne). 2019 Mar 1;10:134. doi: 10.3389/fendo.2019.00134 (PMC6407429; doi:10.3389/fendo.2019.00134)
Supplement: Supplemental Table 1 — Oligonucleotides used for qPCR. [file Table_1.DOC]

Supplemental Table 1. Oligonucleotides used for qPCR

| **Gene** | **Forward sequence (5’-3’)** | **Reverse sequence (5’-3’)** |
| --- | --- | --- |
| *Slc5a5 (Nis)* | *GATCCCCAGTTCTGGAATGGA* | *AGAGATAGGAGATGGCGTAAAAGG* |
| *Tpo* | *ACAGTTCTCCACGGATGCACTA* | *GGCAAGCATCCTGACAGGTT* |
| *Tg* | *CTCAGGACGATGGGCTTATCA* | *GTTCGGCCTTGGCTTTCTTC* |
| *Tshr* | TCGAGACTCACCTGAAGACCATT | TCGCTGCAGAGTGGCATCTAT |
| *Pax8* | CCAGTGGACAGGGCAGCTAT | AATACTCACTTCCTGCCACCAT |
| *Nkx2-1 (Ttf1)* | AGTGAGGAGGATGTCGTCGAA | CCATGAAGCGGGAGACTGTAA |
| *Lin28a* | TGTATTGGGAGTGAGCGACG | CAGTTGTAGCACCTGTCTCC |
| *Lin28b* | TGATAAGTCGGGAGGGAAACC | CATGAATAGTTTGCTTTGGTGTACAA |
| *Oct4 (Pou5f1)* | AGTCCCTAGGTGAGTCGTCC | TGGAAGCTTAGCCAGGTTCG |
| *Sox2* | AGGGCTGGGAGAAAGAAGAG | GGAGAATAGTTGGGGGGAAG |
| *Nanog* | ACACACCCACCCTACTCCAT | ACGATACACAGTGCACACCA |
| *FAM83F* | TGTCAGGACCTGCAGCTCACT | TCCCCATGGGCATGTAGAAG |
| *Rpl19* | CCAATGAAACCAACGAAATGG | TCAGGCCATCTTTGATCAGCTT |
